# Assisted transcriptome reconstruction and splicing orthology

**DOI:** 10.1186/s12864-016-3103-6

**Published:** 2016-11-11

**Authors:** Samuel Blanquart, Jean-Stéphane Varré, Paul Guertin, Amandine Perrin, Anne Bergeron, Krister M. Swenson

**Affiliations:** 1 LaCIM, Université du Québec à Montréal, Montréal, Canada; 2 LIRMM, CNRS - Université de Montpellier, 161 rue Ada, Montpellier, 34392 France; 3 Inria, Université de Lille, Lille, France; 4 Université de Lille, CNRS, Centrale Lille, Inria, UMR 9189 - CRIStAL, Lille, France; 5 IBC Institut de Biologie Computationnelle, Montpellier, France; 6 Département de mathématiques, Collège André-Grasset, Montréal, Canada; 7 Institut Pasteur, Microbial Evolutionary Genomics, CNRS, UMR3525, and Hub Bioinformatique et Biostatistique, C3BI, USR 3756 IP CNRS, Paris, France

**Keywords:** Transcriptome prediction, Splicing orthologs, Eukaryotes

## Abstract

**Background:**

Transcriptome reconstruction, defined as the identification of all protein isoforms that may be expressed by a gene, is a notably difficult computational task. With real data, the best methods based on RNA-seq data identify barely 21 % of the expressed transcripts. While waiting for algorithms and sequencing techniques to improve — as has been strongly suggested in the literature — it is important to evaluate *assisted transcriptome prediction*; this is the question of how alternative transcription in one species performs as a predictor of protein isoforms in another relatively close species. Most evidence-based gene predictors use transcripts from other species to annotate a genome, but the predictive power of procedures that use exclusively transcripts from external species has never been quantified. The cornerstone of such an evaluation is the correct identification of pairs of transcripts with the same splicing patterns, called *splicing orthologs*.

**Results:**

We propose a rigorous procedural definition of splicing orthologs, based on the identification of all ortholog pairs of splicing sites in the nucleotide sequences, and alignments at the protein level. Using our definition, we compared 24 382 human transcripts and 17 909 mouse transcripts from the highly curated CCDS database, and identified 11 122 splicing orthologs. In prediction mode, we show that human transcripts can be used to infer over 62 % of mouse protein isoforms. When restricting the predictions to transcripts known eight years ago, the percentage grows to 74 %. Using CCDS timestamped releases, we also analyze the evolution of the number of splicing orthologs over the last decade.

**Conclusions:**

Alternative splicing is now recognized to play a major role in the protein diversity of eukaryotic organisms, but definitions of spliced isoform orthologs are still approximate. Here we propose a definition adapted to the subtle variations of conserved alternative splicing sites, and use it to validate numerous accurate orthologous isoform predictions.

**Electronic supplementary material:**

The online version of this article (doi:10.1186/s12864-016-3103-6) contains supplementary material, which is available to authorized users.

## Background

The knowledge of all protein isoforms that may be expressed by a gene is fundamental. Recently, several computational methods have been proposed for transcriptome reconstruction that use RNA-seq data for exon identification, and expression levels data for transcript assembly [1, 2]. While exon identification performs quite well, transcript assembly remains difficult for complex transcriptomes. As shown in [2], the best-performing computational methods identified at most 21 % of spliced protein-coding transcripts from *H. sapiens*, and transcript detection remains low even with very high sequencing coverage, leading the authors to conclude that improved results would have to wait for technological advances. Those findings were confirmed by several other studies [3–5], that include methods recently developed such as StringTie [6].

Given these limitations of *ab initio* transcript prediction, it is natural to investigate *assisted transcriptome reconstruction*, in which the knowledge of transcript structures is transferred from one species to another: since transcripts provide a road-map of the successive links between exons, it should be possible to distinguish transcripts that may be expressed from those that may not, by analyzing the sequence of the target genes.

An essential problem is the assessment of the predictions. Confirmation of the prediction that “transcript *t* may be expressed in the target species” is only possible through experimental validation, which can be a long and costly process, since many transcripts are detectable only in specific cells, under specific conditions [7]. Gene predictors that make explicit use of external evidence to predict eukaryotic gene structure have been around since the turn of the century [8], and with the discovery of alternative transcripts in recent years, hundreds of thousands of predicted isoforms are now available in databases [9]. For example, as of June 2016, *Felis catus* had 32 842 predicted isoforms in the RefSeq database, of which 365 are confirmed, and *Canis lupus familiaris* had 45 430 predicted isoforms of which 1 644 are confirmed. These predictions were made by the Gnomon predictor, described in an unpublished paper [10], and whose performance is unknown, especially for predictions that rely exclusively on external evidence.

In order to evaluate the predictive power of transcript annotation transfer, it is necessary to identify *splicing orthologs*, loosely defined as transcripts from ortholog genes with similar splicing patterns. Zambelli et al. [11], introduced the concept and gave three possible definitions that yielded estimates ranging from 31 to 86 % for the percentage of human transcripts that have a splicing ortholog in mouse. This wide range of estimates is an indicator of how the concept is still a work-in-progress. A subsequent paper of Fong et al. [9] simplifies the definition of splicing orthologs as “…all protein-coding exons in the two proteins can be paired with 90 % overlap in lengths of both exons.”. This is clearly an over-simplification that ignores, among other things, nearby alternative donor or acceptor splice sites [12].

In this study, we revisit the concept of splicing orthology and we give a comprehensive assessment of the performance of assisted transcriptome reconstruction using the human to predict mouse, and *vice versa*.

## Methods

Our experimental set-up is based on a procedural definition of splicing orthologs that is concurrently implemented by two procedures. Our *predictor* uses transcripts from a known species to predict transcripts in a target species, and for evaluation purposes, identifies putative pairs of orthologous transcript isoforms based solely on nucleotide sequence evidence. Our *controller* identifies putative pairs of orthologous protein isoforms between human and mouse using amino acid sequences and positions of exon junctions.

### Splicing orthologs

Transcripts from orthologous genes with differing splicing patterns could have easily identifiable differences in the number or identity of their exons. However, defining splicing orthology can be more difficult due to the presence of alternative donor and acceptor splice sites, where exons are elongated or truncated, often by a very few nucleotides. Within a gene, these exon isoforms [13] overlap and have different exon-intron border(s).

Across species, we define *orthologous exon isoforms* as orthologous exons that have conserved exon-intron borders. Using this concept, *splicing orthologs* are defined as transcripts of orthologous genes, whose exons are orthologous exon isoforms that appear in the same order in each gene, and that code for similar proteins.

In the ideal situation where all splicing orthologs are conserved across species in one-to-one orthologous gene pairs, all exons — with their flanking intronic sequences — should be best reciprocal hits, and the alignments should preserve exon-intron borders. Each protein would have a *perfect match* that should be a *unique* best reciprocal hit, the alignments should preserve the positions of exon junctions, and be without gaps in the near vicinity of junctions.

### The predictor

Given a pair of orthologous genes, the first task of the predictor is to establish a common reference sequence [14] for the set of all transcripts in both the human and mouse. Our solution is based on the concept of *blocks*, which are alignments of human and mouse exon segments that are either contained in, or disjoint from, each human or mouse transcript.

**Fig. 1:**
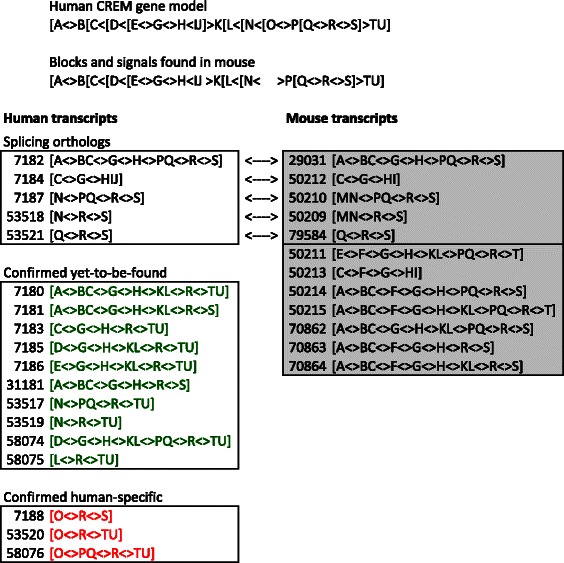
Gene models and transcripts. In the CCDS Database [18], as of June 2016, there were 18 CREM human and 12 Crem mouse transcripts with unique splicing patterns. The common reference sequence has blocks labeled from A to U. The human gene model is at the top of the figure, followed by the sub-sequence of blocks and signals found in the mouse. The block representation and CCDS number is given for each transcript. Note that block O does not exist in the mouse gene, and block F is not in the human model, since block F is not found in any known human transcript. Of the 18 human transcripts, 15 are executable – meaning that they could be expressed –, and three are not (in *red*) because they use block O. The executable transcripts are further classified as found (5 of them, in *black*) and paired with a mouse transcript, or yet-to-be-found (10 of them, in *green*). All these predictions are confirmed by the controller. Since 5 mouse transcripts are correctly identified, and 12 mouse transcripts are currently known, the predictor successfully identifies 5/12, or 42 % of the mouse transcripts. As more mouse transcripts are discovered, this proportion may increase with future releases of the CCDS database

Each block has a label and can be flanked by *signals*: start and stop codons, donor and acceptor splice sites. Using block labels, the symbols ‘[’ and ‘]’ to indicate start and stop codons, and ‘<’ and ‘>’ to indicate the intronic part of donor and acceptor splice sites, each transcript can be represented as a string. The *gene model* of a set of transcripts is the ordered string of its blocks and signals, and a *donor block* (resp. *acceptor block*) is a block that has a donor signal at its 3’ end (resp. an acceptor signal at its 5’ end) in the gene model. Figure 1 presents an example with the CREM and Crem orthologous genes of human and mouse [Accession numbers ENSG00000095794 and ENSMUSG00000063889].

To ensure internal exons best reciprocal hit property, every human and mouse sequence in a block is Blasted, in the orthologous gene, using flanking genomic sequences of length 20. Further details on the predictor algorithm can be found in Additional file 1.

The fact that all blocks can be found by a Blast search allows the predictor to simulate a pure predicting mode, that does not have any prior knowledge of the target species transcripts. Given a gene model *M*, the predictor reports all blocks and signals that were found in the target gene, and reports its successes as a substring *S* of the gene model.

This paper predicts in *flexible* mode [2, 11], where orthologous transcripts must have the same splicing pattern, but the first and last exons are only required to have orthologous internal exon-intron borders. A transcript is said to be *executable* by the target gene if its donor and acceptor blocks are in *S*. The primary task of the predictor is to classify transcripts as executable or not executable.

In the simulation context of this paper, a secondary task of the predictor is to classify executable transcripts of the known species into *found* or *yet-to-be-found*, using the transcripts of the target species. When a known transcript *k* has a match *t* in the list of transcripts of the target species, the predictor reports a pair of putative ortholog transcripts (*k,t*). Thus the output of the predictor is a list of transcript pairs, a list of yet-to-be-found transcripts, and a list of non-executable transcripts.

### The controller

The controller outputs a list of putative human/mouse protein ortholog pairs whose underlying transcripts have the same splicing patterns. For each gene, the controller constructs a list of candidate pairs, that are unique best reciprocal hits among protein isoforms of the gene with the same number of exons.

**Fig. 2:**
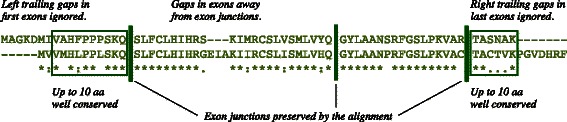
Alignment validation. Requirements for candidate protein sequences to be included in the controller list

Alignments are done using a semi-global exact alignment [15], that does not penalize left or right trailing gaps, and are scored with the Blosum62 scoring matrix. For a candidate pair to be accepted, the alignment must preserve all exon junction positions, and be without gaps in the near vicinity (*v*=2 amino acids) of junctions, as illustrated in Fig. 2. Insertions and deletions within an exon are allowed. The parameter *v* was experimentally determined to maximize the agreement between the predictor and the controller: there is no easy solution since gaps at or near exon junctions can be the result of insertions or deletions at the protein level, or evidence of alternative donor or acceptor sites.

Finally, the right segment of the alignment of the first exon of each transcript, and the left segment of the alignment of the last exon, are tested for conservation by requiring non-negative Blosum62 scores, over a maximum of 10 amino acids. This last step is necessary because the position of the first (or last) exon junction can be preserved even when the first (or last) exons are different.

### Validation

We implemented a co-validation process between the two procedures, with the principle that both procedures should agree on their conclusions: disagreements are treated as errors.

The output of the predictor is compared to the controller list:

- A transcript pair found by the predictor that also belong to the controller list is labeled *splicing orthologs*.
- A transcript *k* classified as yet-to-be-found, for which the controller list does not contain any entry with transcript *k*, is labeled *confirmed yet-to-be-found*.
- A transcript *k* classified as non-executable, for which the controller list does not contain any entry with transcript *k* is labeled *confirmed species-specific*.

Predictions that are not confirmed are called *unresolved*. There exist three types of unresolved predictions:

- Found by the predictor, but not contained in the controller list.
- Yet-to-be-found by the predictor, but contained in the controller list.
- Species-specific by the predictor, but contained in the controller list.

The number of each of these types of errors are reported and discussed in the next section.

## Results and Discussion

All 15 513 one-to-one ortholog gene pairs with at least one CCDS isoform in human and in mouse were selected in the Ensembl database [16], as of 07/30/2015, the date of CCDS Release 19. Of these, the predictor could analyze 14 992 pairs of genes, totaling 24 382 human and 17,909 mouse transcripts with more than one exon. The 521 genes rejected at this stage had homology problems such as duplicated or rearranged exons, or were not one-to-one orthologs. The controller list computed on this set has 11 904 pairs of putative protein ortholog pairs. When transcripts from the same species are splicing orthologs, thus having the same splicing pattern, both procedures keep the isoform with the smallest CCDS accession number.

The first experiment predicts mouse transcripts using human as the known species. In this case, there are about 36 % more transcripts in the known species than in the target species. The second experiment mirrors the first, with the known species (mouse) having fewer transcripts than the target species (human). We also partitioned the complete set of predictions according to the CCDS release in which the transcript of the known species first appears.

We looked at the complete set of predictions, and also at subsets ordered by their CCDS release numbers. The results are analyzed from three different perspectives: What is the difference between early and late predictions? If we had made these predictions in the past how long would it have taken to confirm them? What is the impact of the size of the predicting set of transcripts? Unresolved cases are discussed at the end of the section.

### Early cohort has good precision and recall values

The predictor and controller analyzed 24 382 human transcripts, and agreed in 23 165 cases (95.0 %). Table 1 gives detailed results of the resolved predictions of mouse transcripts using human transcripts. Precision and recall are computed using the definitions established in [17] for gene structure prediction programs, called *specificity* and *sensitivity* in the original paper.

**Table 1:** Mouse transcripts as predicted by human transcripts

	Predictions	10/2006	01/2011	07/2015	
		*C*_1_	*C*_2_	*C*_3_	All
*a*	Splicing orthologs	7 307	3 000	815	11 122
*b*	Confirmed yet-to-be-found	1 332	1 631	3 006	5 969
*c*	Confirmed human-specific	1 644	1 793	2 637	6 074
	Unresolved	612	294	112	1018
*d*	Total number of analyzed transcripts	11 002	6 797	6 583	24 382
*b*/*d*	Proportion of yet-to-be-found transcripts	0.12	0.24	0.46	0.24
*c*/*d*	Proportion of human-specific transcripts	0.15	0.26	0.4	0.25
	Mouse reality				
*e*	Splicing orthologs, by mouse cohorts	7 024	3 454	644	11 122
*f*	Known mouse transcripts	9 486	6 348	2 075	17 909
	Statistics				
*a*/(*a*+*b*)	Precision	0.85	0.65	0.21	0.65
*e*/*f*	Recall	0.74	0.54	0.31	0.62

The precision value is the proportion of splicing orthologs over the total number of *positive* predictions, defined here as the sum of the number of splicing orthologs and and the number of confirmed yet-to-be-found transcripts. For the first experiment we have a precision of (11122)/(11122+5969)=0.65.

The recall value is defined in the literature as the proportion of correctly identified isoforms with respect to the number of real isoforms, and is computed using the number of splicing orthologs over the number of known mouse transcripts, and is (11122)/(17909)=0.62.

The same precision and recall computations were done after partitioning the predictions in three time-stamped cohorts, each ending at a CCDS Mouse release. Cohort *C*_1_ contains transcripts known at Release 2 (10/2006); cohort *C*_2_ contains transcripts added to CCDS between Releases 3 and 7 (01/2011); cohort *C*_3_ contains transcripts added to CCDS between Releases 8 and 19 (07/2015). Note that the number of splicing orthologs is different for human and mouse cohorts, but they all add up to the total of 11 122 transcript pairs: indeed, transcripts *k* and *t* of a pair (*k,t*) may belong to different cohorts.

For the first cohort, the precision and recall are, respectively, 0.85 and 0.74. Meaning that 74 % of the transcripts of the first mouse cohort are correctly predicted, which is more than three times the 21 % obtained by the purely computational methods tested in [2]. Precision and recall fall with subsequent younger cohorts, and this phenomenon is discussed in the next section.

Table 1 also shows the large increase in the proportion of yet-to-be-found transcripts, from 0.12 in cohort *C*_1_ to 0.46 in cohort *C*_3_. This increase was expected, and is discussed further in the next section.

On the other hand, the increase of the proportion of human-specific transcripts, from 0.15 in cohort *C*_1_ to 0.4 in cohort *C*_3_ was unexpected. Possible explanations would be that the first cohort is dominated by highly expressed ubiquitous isoforms that were detected early; and/or that species-specific transcripts are less expressed, and are found later; and/or that recent sequencing experiments are more focussed on exploring the difference between mouse and human.

### Performances get better over time

Here we analyze the evolution over time of the number of splicing orthologs, for a fixed number of positive predictions, which drives the evolution of precision values. We restricted this analysis to genes that have a large number of isoforms, defined here as genes that have at least two different CCDS isoforms in human and in mouse. This is a subset of 4253 positive predictions of the first experiment. Each cohort of this subset has respectively, 1464, 1404 and 1385 positive predictions.

**Fig. 3:**
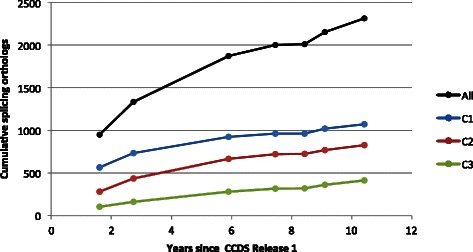
Evolution of the number of splicing orthologs. These curves show the growth, over the years, of the number of known splicing orthologs among the subset of orthologous genes that has at least two different isoforms for human and for mouse in the CCDS Release 19. Each data point corresponds to a CCDS release of mouse transcripts: releases 2, 4, 7, 10, 13, 16 and 19. The *black* curve shows the growth of the whole subset; the *blue* curve shows the growth of splicing orthologs whose human transcript was known in 2006; the *red* curve shows the growth of splicing orthologs whose human transcript was discovered between 2006 and 2011; and the *green* curve shows the growth of splicing orthologs whose human transcript was discovered since 2011

Figure 3 plots the cumulative number of known splicing orthologs, at the time of CCDS Releases 2, 4, 7, 10, 13, 16 and 19, each of these being a release of mouse transcripts. (The data used is available in Additional file 2.) The black curve corresponds to all known splicing orthologs, and shows a healthy growth over time – more than doubling its size – with a short standstill due to the very low number of new transcripts (*n*=159) added in Release 13. This means that, with the same set of 4253 positive predictions, we can expect the precision to increase with each new Mouse release.

The three colored curves of Fig. 3 correspond to the partition of the set of predictions in cohorts *C*_1_ to *C*_3_. They start at different heights, with cohort *C*_1_ leading, but the fact that all curves have a similar shape, notably in the last half of the time interval plotted, was highly unexpected. Indeed, since the number of positive predictions is fixed for each cohort, the number of cumulative confirmed predictions must eventually become almost flat, with less and less predictions being confirmed. We expected such a phenomenon, especially for cohort *C*_1_ whose precision is currently 74 %, but the saturation effect is barely perceptible in this subset.

A similar shape in cumulative curves indicates a similar absolute growth over time. This means that, for the last four releases, all cohorts have roughly the same number of new splicing orthologs. Given that all three have similar numbers of positive predictions, we expect the number of splicing orthologs to grow significantly in future CCDS releases, at least for genes that have a large number of isoforms.

### The number of known species transcripts influences both precision and recall

Table 2 presents the results of the prediction of human transcripts using mouse transcripts. All the trends observed in Table 1 are in the same direction, but with varying intensity. Notably the precision values are higher, which is consistent with the expectation that a smaller set of predicting transcripts yields better chances of confirming a prediction. On the other hand, the recall values are lower, since the number of transcripts to identify in human is much larger.

**Table 2:** Human transcripts as predicted by mouse transcripts

	Predictions	10/2006	01/2011	07/2015	
		*C*_1_	*C*_2_	*C*_3_	All
*a*	Splicing orthologs	7 024	3 454	644	11 122
*b*	Confirmed yet-to-be-found	729	1 103	751	2 583
*c*	Confirmed mouse-specific	1 074	1 322	595	2 991
	Unresolved	554	390	70	1014
*d*	Total number of analyzed transcripts	9 486	6 348	2 075	17 909
*b*/*d*	Proportion of yet-to-be-found transcripts	0.08	0.17	0.36	0.14
*c*/*d*	Proportion of mouse-specific transcripts	0.11	0.21	0.29	0.17
	Human reality				
*e*	Splicing orthologs, by human cohorts	7 307	3 000	815	11 122
*f*	Known human transcripts	11 002	6 797	6 583	24 382
	Statistics				
*a*/(*a*+*b*)	Precision	0.91	0.76	0.46	0.81
*e*/*f*	Recall	0.66	0.44	0.12	0.46

### Unresolved cases

Unresolved cases are the results of disagreements between the predictor and the controller. Both procedures are experimental, in the sense that they depend on parameters and thresholds, and adjusting these values was done with the goal of maximizing agreement, which is 95.0 % when human transcript is used to predict mouse transcripts.

There are 1018 unresolved cases in total, since most of them appear in both experiments. Of them, 369 ortholog pairs are found by the predictor but not by the controller and often reflect a built-in stringency of the controller. In order to distinguish subtle modifications in the regulation of alternative transcription, such as *nagnag* alternative transcripts [12] that add or delete one amino acid next to an exon junction, the controller rejects every pair that contains deletions in near junctions. Using the nucleotide sequences, the predictor has an advantage in deciding whether such insertion or deletion is a true mutation. The controller can also detect frame-shifts, where a single mutation can cause two dissimilar proteins in different species. This is currently not verified by the predictor.

There are 649 instances of pairs included in the controller list for which one of the transcript is predicted yet-to-be-found or species-specific by the predictor. They are due to pairs incorrectly included in the controller, or to the built-in stringencies of the predictor. In the case of the controller, the main source of errors is the presence of conserved alternative exons of the same length, yielding two possible isoforms *A* and *B* in the human, and *A* ^′^ and *B* ^′^ in the mouse. If the current state of the database contains *A* and *B* ^′^, the controller will pair them, but the predictor will correctly detect that the transcripts differ by one exon. When three of the four isoforms are present, the unique best-hit property of the controller construction will resolve the conflict. A second source of errors from the controller is the presence of very small first or last exons, sometimes as small as 1 nucleotide.

## Conclusion

We gave a rigorous high level definition of splicing orthology and implemented it with a dual predictor/controller. We applied the methods to the CCDS human and mouse sets of isoforms and classified them into pairs of splicing orthologs.

We also showed that, for the prediction that could have been made eight years ago, human transcripts would have correctly predicted 7 024 of the 9 486, thus 74 % of the known mouse transcripts at that time. We showed that this percentage is a lower bound, since predictions for that cohort are still being confirmed with each new release of the CCDS database, driven by the discovery of predicted isoforms of genes that have a large number of isoforms.

Our list of 11 122 confirmed splicing orthologs is available in Additional file 3, together with their common block representations. It is intended as a benchmark for predictors, and as a data source for researchers interested in studying the conservation of alternative splicing.

## Additional files

Additional file 1 PredictorAlgorithm. Description of the prediction algorithm. (PDF 97 kb)

Additional file 2 Results.tar. Compressed folder containing csv files listing the results. (GZ 3942 kb)

Additional file 3 **TableS1**. Data for Figure 3. (PDF 21 kb)
