# Supplementary material for: Assisted transcriptome reconstruction and splicing orthology
Source: BMC Genomics. 2016 Nov 11;17(Suppl 10):786. doi: 10.1186/s12864-016-3103-6 (PMC5123294; doi:10.1186/s12864-016-3103-6)
Supplement: Supplementary file 1 — PredictorAlgorithm. Description of the prediction algorithm. (PDF 97 kb) [file 12864_2016_3103_MOESM1_ESM.pdf]

Description of the predictor algorithm

Given a pair of orthologous genes and a *gene model* built from a set of transcript isoforms from the known species, the algorithm aims at identifying orthologous functional *signals* and orthologous *blocks* shared between the genes of the *known* and *target* species. The algorithm outputs a *gene model* for the target species, defined as a string of predicted signals and blocks. Given this target gene model, we check whether transcripts of the known species are *executable* or not (that is, *could be expressed* or not) in the target gene model.

Detection of block homology

To detect the presence of orthologous signals and blocks of the known species in the target species, one needs to compute alignments of each block of the known species gene model with the genomic sequence of the target species gene.

Assuming that orthologous exons would generally have identical lengths, blocks are first searched using the Blast ungapped configuration. If the quality of the alignment is low, a gapped search with Blast is performed, with a seed of size 7.

Blocks are flanked on both sides by a context of 20 nucleotides. We only look at the best Blast hit and the following filters are applied in order to select an alignment: an e-value lower than 10^{-5} for blocks longer than 20 nucleotides, and lower than 10^{-1} otherwise. To select an ungapped Blast hit, we also require that the hit covers all the block from its beginning to its end, including the di-nucleotide motifs of its splicing sites. When an ungapped Blast hit is selected, no gapped Blast hit is considered.

The selected alignment is assigned to the block and its flanking signals.

Assignment of orthology to actual signals

Optionally, it is possible to provide to the predictor annotated transcripts for the target species. Then, the algorithm also takes as input a gene model for the target species gene. In this case, blocks in the target gene model are aligned, as described in the previous section, against the known species gene sequence. Given the two gene models and the selected alignments, we look for pairs of orthologous actual signals (signals in genomic sequences).

Given an actual signal in a gene model (either known or target), we test whether it is aligned or not against an actual signal in the orthologous gene counterpart. If so, we conclude that the two actual signals are orthologous. Moreover, we insure that all pairs of orthologous actual signals appear in a colinear order in both genes. Orthology status of the remaining actual signals will be solved in the next step.

Note that we do not require a reciprocal best Blast hit to assess the orthology. However, note that if two signals remain orthologous when the roles of known and target species are reversed, this implies a reciprocal best hit between them. Consequently, if a known transcript is executable by the target gene model and found in the target transcript set, and the same is true of the known gene model and known transcript set, then all of their blocks participate in reciprocal best hits.

Note moreover that, since existing blocks and signals are considered for the target gene, the current step may add predicted blocks and signals to the known gene model.

Prediction of signals in the target species

For each remaining signal in the known gene model we aim to decide if it:

- has no orthologous counterpart (no Blast hit),
- has no comparable orthologous signal, but the signal has an *image* site in the alignment,
- has a possible orthologous signal displaying a relevant nucleotide motif.

If a signal does not belong to an alignment, or if it is aligned against a gap, then the signal is annotated as having no orthologous counterpart. The signal is annotated as being orthologous to the corresponding site in the target sequence (called a predicted signal) if this site's position and sequence in the alignment are correct, otherwise, the signal is annotated as having an *image*. (i) The position is correct if it occurs between the preceding and the following pair of orthologous signals (if any) in the known and target gene models. This insures that the colinearity of orthologous signals are preserved. (ii) The signal alignment is correct if the image site contains the same nucleotides in the same order. This applies to the `ATG` start codon, as well as to `GT` donor and `AG` acceptor splice sites, and other less common splice site motifs. In the case of the `TAA`, `TAG`, and `TGA` stop codons, any match to any stop codon yields a predicted signal. Finally, a donor or acceptor site is retained as a valid prediction only if it is not immediately flanked with gaps in the alignment.

At the end of this step, all actual signals of the known gene have received an orthologous counterpart site: either absent, present but not functional, or present and functional. Present and functional sites are predictions belonging to the target gene model.

If the annotated transcripts for the target species have been provided, the same work is done with remaining actual signals in the gene model of the target species. This will thus possibly introduce predicted signals and blocks in the known gene model.

Refinement steps

Some signals could be inaccurately aligned by Blast resulting in pairs of signal orthologs that we could have missed.

The local and gapped Blast alignment may have not reached one end of a block, while it covers the other end. The block's covered end allows the inference one of its flanking pair of orthologous signals, but the uncovered end makes signal detection on that side more difficult. In this case, we compute a global alignment of the block pair; this alignment is analyzed as described in the previous step. If it includes the missed signals, each would receive an image site, which would eventually be aligned and orthologous.

We next try to recover missed orthology between small blocks, which cannot be aligned by Blast with significant e-values. For each interval between consecutive pairs of orthologous sites, we seek pairs of blocks, one in each gene model, having no orthologous counterparts in the other gene model. When two such blocks display identical lengths and equivalent functional structures (*i.e.* flanked by the same signals), then the method declares that the signals flanking those blocks are orthologous.

Block orthology and naming

Given consecutive pairs of orthologous sites (*i.e.* actual signal, predicted signal, signal image) in both gene models, let's consider the two sets of blocks contained between these sites. If these two sets contain one block each, and if both are exon blocks, then the blocks are declared orthologous and they receive a common block name. If one or both of the two sets contain more than one coding exon block, then these exons have no orthologous counterparts, and they each receive a specific block name.

At the end of this algorithm, the *gene model* of the known species has been annotated with an orthology relationship on all of its signals and blocks, and a common labelling of the blocks across the pair of orthologous genes. These relationships and labels define the gene model of the target species.

Flexible gene model

In the context of this paper, the predictor first classifies transcripts of the known gene as *executable* or not by the target gene model. Executable transcripts of the known species are then classified into *found* or *yet-to-be-found*, if they belong or not, respectively, to the set of known isoforms of the target species gene. The predictor could take into account actual transcripts for the target species, as described in the first section.

To achieve this goal, we use the common block labeling inferred for the known and target sets of transcripts. While all transcripts of the known (respectively target) species could be written using the labels of the known (respectively target) gene model, only part of the known transcripts could be written using the labels of the target gene model (as shown in Figure 1). Those known transcripts are said to be *executable* by the target gene model.

In this work, we use a *flexible* scheme for the execution of known transcripts in the target gene model, where first and last exons are only required to have orthologous internal exon-intron borders. This implies that isoforms having no orthologous start or stop codon, but sharing orthologous first and last exons, are considered orthologous.

Finally, as target transcripts can all be written using the labels of the target gene model, they can be directly compared to the executable known transcripts. This final comparison allows the algorithm to decide whether an executable known transcript is *found* within the actual target transcripts set, or not. In the latter case, the executable known transcript is called *yet-to-be-found*, indicating that it could be a true but not yet observed isoform of the target species gene.
