# Supplementary material for: Assisted transcriptome reconstruction and splicing orthology
Source: BMC Genomics. 2016 Nov 11;17(Suppl 10):786. doi: 10.1186/s12864-016-3103-6 (PMC5123294; doi:10.1186/s12864-016-3103-6)
Supplement: Supplementary file 3 — TableS1. Data for Figure 3. (PDF 21 kb) [file 12864_2016_3103_MOESM3_ESM.pdf]

			Number of splicing orthologs			
Release	Date	No. Sequences	C1	C2	C3	All
2	10/10/06	13374	565	282	104	951
4	11/28/07	17707	734	437	162	1333
7	01/24/11	22187	923	666	283	1872
10	08/14/12	22934	963	721	317	2001
13	08/05/13	23093	963	725	321	2009
16	04/07/14	23880	1019	770	363	2152
19	07/30/15	24834	1073	826	413	2312
Positive predictions			1464	1404	1385	4253
